# Portable Dental Chairs and Their Role in Assisting the Community Outreach Program- a Qualitative Approach

**Published:** 2013-05

**Authors:** Firoozeh Nilchian, Ali Sahlabadi, Masoomeh Skini

**Affiliations:** 1 Torabinejad Dental Research Center and Department of Dental Public Health, Isfahan University of Medical Sciences, Isfahan, Iran; 2 The College of Law, Braboeuf Manor, Guildford, UK; 3 Student of Dentistry, Student Research Center, School of Dentistry, Isfahan University of Medical Sciences, Isfahan, Iran

**Keywords:** Mobile Health units; Health Services Accessibility; Oral Helth

## Abstract

**Objective:** Certain conditions limit the independence of deprived people. However, those conditions do not limit the right of these people to receive dental care. Portable dental units may make it possible for the deprived and elderly to receive the care they deserve in more areas. This study aimed to explore dentists’ perceptions of the use of portable dental units in community outreach programs as a precursor to our quantitative study in the future.

**Materials and Methods:** Qualitative semi-structured interviews were conducted by one interviewer, seven dentists, two specialists, and 11 final-year dental students who had experience with portable dental units in Isfahan, Iran. The qualitative research method was chosen first due to the rarity of information readily available about the units and second due to their specialist application; with which only certain members of the dental community have experience.

**Results:** Participants described a range of observations they had made whilst using the portable dental units in different locations. The qualitative data were primarily concerned with the range of ideas the interviewers distinguished as relevant when describing the portable dental units. The predominant view most noted within interviews was that the portable units are very useful for the community outreach programs, with two subcategories of serviceability and access to oral health. Other factors mentioned were the competence of the portable dental unit and the factors affecting each individual patient.

**Conclusion**: Our findings illustrate a number of features that may enhance the reputation of portable dental units, due to their useful nature.

## Introduction

One of the limitations of the dental profession has been its dependency on fixed equipment such as the dental units or chairs. 

The dental units generally need connecting to electrical, as well as drainage and plumbing systems.Transportation to the dental office is cited by many of the elderly as a barrier to dental treatment [1].

**Fig1:**
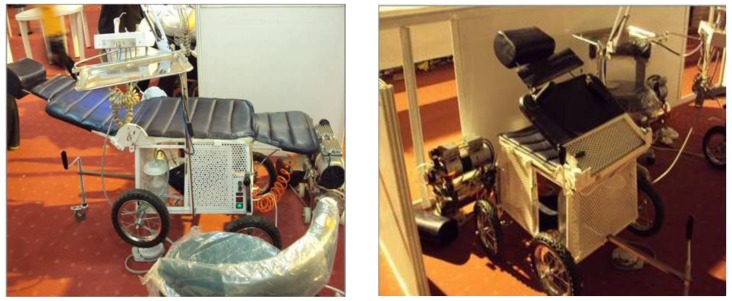
The portable dental units designed in Isfahan dental university

Mobile (bus dental unite) or portable services eliminate the transportation barrier by bringing the service to the client [2]. The portable dental chair will provide greater assistance to disabled clients living in those out of reach places [3]. They make it possible for the elderly to receive the care they deserve [4,5]. The mobile and portable dental services will also enable care for the elderly in their homes or care facilities. The demand for dental care will continue to increase. The elderly population is rapidly growing in the world. It is estimated that 3.5 million US elderly people will be living in long-term care facilities by year 2030 [6].In some situations, transport infrastructure may also restrict access to care. There are some areas in Iran that are inaccessible when roads are not passable, such as the mountainous villages in and around Fereydoon-shahr. 

In response to these limitations, a prototype portable dental chair/unit was designed by Isfahan dental school. The basic system includes an operator light source, an examination kit, a portable head rest, and a first aid kit for dental purposes (Fig1).These portable dental units used have a rotary instrument and an operator light fixture that is packed in two cases (Fig1).

More complex portable units include a vacuum canister, ultrasonic scaler, radiographic equipment along with compressors for air-water syringes and high- and low-speed handpieces [7]. This equipment is stored and transported in durable boxes and cases [8].

The rationale for these chairs is to allow provision of simple filling and basic preventative treatments such as fluoride therapy and fissure sealants, but they remain largely unevaluated. Therefore, the aim of this study was to explore dentists’ perceptions of the use of portable dental units in community outreach programs.

## MATERIALS AND METHODS

Qualitative semi-structured interviews were conducted by one investigator with seven dentists, two specialists and 11 final-year dental students. 

Interviewees had been picked from the out-reach program at Isfahan dental university, and also at the Isfahan trade fair. All had prior experience with portable dental units. Recruitment was via letters to all students and dentists working with portable dental units. 

Interviews were loosely structured and comprised open-ended questions related to the area to be explored.

**Fig 2:**
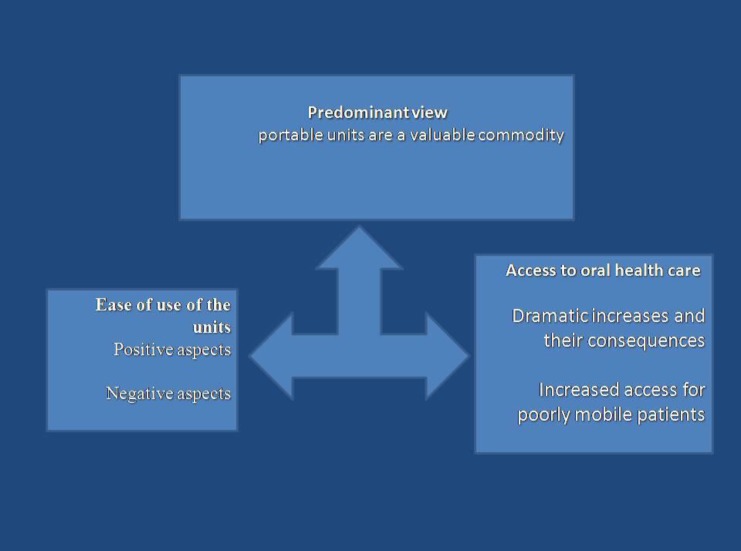
Categories and subcategories of professional views

Based on preliminary discussions between the researchers, dentists, specialists and students, purposive sampling was used to recruit participants to capture a range of experiences such as the practice setting, length of time qualified, role in the practice or service, age and gender. 

Sampling continued until ‘saturation’ was achieved when no new codes appeared in the data [9]. Interviews were audio-recorded and transcribed. The principle approach was qualitative content analysis. First data were analyzed using line by line coding. These codes were then grouped. After this initial familiarization with the data, the groups were revised to form sub-categories and categories originated in the data. Finally, the categories were grouped into themes that provided insights into, and explanations of factors that might influence the success of treatments such as fissure sealants using portable units. 

## RESULT

Participants demonstrated a range of perceptions as to the type of experiences they had using portable dental units in different types of locations. 

The experiences could be grouped under two broad categories: ease of use of the units and increasing access to oral health care. Each of these categories incorporated a series of sub-categories (Fig 2). 

However, a predominant view across most of the interviews was that the portable units are a valuable commodity. 

***The ease of use of the portable units***

This section details a variable theme in the ease of use of portable units divided into positive and negative features.

Among the negative features, some dental operators' expressed the view that there were limitations with the chairs.

*‘* *It was hard to adjust the chair for some positions, especially for the upper* *teeth, there were only three positions. It is not like a normal unit that can be adjusted in different positions'*

*(Dental Students)*

From this perspective we felt that there were some problems especially regarding adjustment of the dental chair. However, some dentists mentioned that working with the portable dental units was easy. 

*‘The portable units were easy to work with, I didn't have a problem with them’*

*(Dentists) (Specialist)*

Dental operators also expressed their views about the rotary systems which include high speed, hand-piece and air-motors 

In particular,

*‘The rotary systems were good; I didn’t find problems for cavity preparation’*

*(Dental Students)*

***Access to oral health care***

Access to dental care was increased in two ways. There was a dramatic increase in access to care for some populations and also an increase in access for patients who might be near but are not able to move.

Dramatic increases in access occurred for small populations restricted by unapproachable terrain (eg mountainous regions). Such areas, without dentists and/or dental equipment were seen by some participants as a good reason to use portable units: 

*‘Having dentists who can come seasonally with their portable equipment could be useful for populations without any dental practitioners of their own.*

*(Dentist)*

*‘There was one hygienist there [Fereydon-shahr] only with forceps to extract teeth. Having portable dental units can provide more treatment options for the populations’*

*(Dental Student)*

As well as inaccessible areas in general, two specific types of communities were mentioned, those were public transport had to be used and for nomadic populations.

*‘Carrying dental equipment in the mountains is improbable by bus; however, we find using these units [portable units] a cost effective and practical solution’*

*(Dental Student)*

*‘The nomadic population who migrate during the summer and winter find implementation of the portable dental units very resourceful’* *(Dentist)*

One category that emerged as a consequence of access were extreme cases that arose from the patients. In some deprived areas it was mentioned that people with poor oral health did not know anything about dentistry or about dentists. In these instances, portable units were very useful in educating and increasing the knowledge of such people about oral health. 

*‘They were very clever children but didn't have any oral health education and their parents would extract so many of their teeth because they were unfamiliar with dentistry. Therefore, the portable unit was very interesting to them' (Dental Students)*

The final category that emerged in relation to access were those patients who were not necessarily distant from the existing clinics, but had mobility problems that prevented them to go to the dentist.

## Discussion

This is the first qualitative study to explore dentists’ perceptions of the use of portable dental units in a community outreach program. The rationale behind the investigation was to gain greater insight into how portable dental unit use could be effective and to identify areas for improvement. Qualitative data are primarily concerned with identifying the range of ideas and categories within the data and are not suited in describing how frequent is a particular view [26]. Deviant case analysis was also undertaken to ensure that any emergent explanations or theories were redefined to embrace all cases [Pope and Mays, 2000]. The emergent categories are neither discrete nor mutually exclusive and are the constructions of the researchers to group and understand the data. Therefore, the results are presented as themes emerged from the data rather than coherent categories described by participants. However, quotes are used to illustrate key categories. Initials are used to anonymize the quotes. Our findings illustrate a number of potentially important areas, considering which may enhance the overall function of the portable dental units. First, the interviewees described the useful nature of the portable units. Access to oral health service was also another point discussed by the interviewees. Overall, the portable unit was seen as a reliable instrument for dealing and assisting regions disconnected from motor vehicles (Fereydoon Shahr). In this study, lack of dentists and dental equipment was seen by some participants as a good reason to use portable units. Portable units seemed to be a reasonable replacement for people with problems accessing dental care. This finding is compatible with earlier research on dentists’ views regarding use of portable units in community outreach programmes [10]. Portable chairs are not dependent upon access to water or pressurized air and only require connection to electrical sockets. Therefore, portable dental units are a substitute yet dynamic solution to dental care in hard to reach places [11-13]. A study published in 1972 claims that 300 clients in a nursing home may be treated in a week with the right equipment and proper coordination [14]. 

A study by Krust and Schuchmang reported that the portable systems provided more care to long-term-care facilities than mobile systems. This may be because portable dental equipment is generally less expensive, more accessible, and less susceptible to mechanical difficulties [15]. 

The findings illustrated by Dane [16] similar to our findings also stated that by working on the mobile dental units, practitioners can "increase the awareness of the practicing dental community”. 

The oral health education that the elderly, children and their families and the long-term-care-facility staff members receive through portable dental units may increase the value placed on preventive dental care [17] and improve the community oral health status [11]. These are compatible with our results.

While advantages and disadvantages to the use of portable systems exist, their implementation provides dental services to people who would otherwise not receive treatment [18]. Our finding supports the results of these studies. Although portable dental units may be used as long-term facilities, disadvantages do exist in using these systems. 

Disadvantages include dependency upon electricity meaning the device is not completely self-sufficient. Portable units may not be appropriate for periodontal treatment or complex oral surgeries. Portable dental systems may also be incapable of delivering all the services provided by mobile dental systems, such as endodontic procedure [19].

Another issue with the use of mobile and portable dental units is the continuity of care that the clients receive. Adequate dental services are not available once the mobile or portable dental units leave the facility [20]. Until permanent dental settings can be implemented in every long-term-care facility, mobile and portable dentistry can provide dental care for the patients with access problems [21]. That is similar to the comments mentioned in this study.

*‘Using dentists in seasonal periods could be useful for the populations without dental equipments and dentist’*

*(Dentist)*

The cost-effectiveness of portable dental units is one of the fundamental reasons for this present study. The results support the idea that while the mobile and portable dental systems are not ideal care solutions for the growing population, they do offer several advantages over traditional dental delivery systems. These dental units decrease the financial transportation and motivational barriers to dental treatment that clients face [22, 23]. They also offer a community outreach program to promote and generate personal satisfaction for the clients and the dental team [24, 25], issues such as time management, treatment options, and continuity of care. 

## CONCLUSION

This study has identified a variety of factors, these factors may influence the way in which the portable dental units are used within the community outreach programs, in particular when describing the specific issues participants of the outreach program experienced when using the units in and around Isfahan province. The competence of the portable units was another key category in the interviewees descriptions; serviceability of the portable units including 1/ human resources and 2/ dental equipment became a key topic for discussion. The overall consensus was the belief that the dental equipment was satisfactory apart from a few faults being described by some interviewees. These faults included the inflexibility and stiffness of the equipment and also the limited dental procedures the dentists could provide with the portable units. 

Efforts to promote portable dental chairs and dental vehicles will be necessary to convince both the public and the government to provide funding for these types of services.
